# SNX11 Deletion Inhibits Dabie bandavirus Infection by Interfering with the Assembly of V-ATPase

**DOI:** 10.3390/pathogens14070677

**Published:** 2025-07-09

**Authors:** Tiezhu Liu, Xueqi Wang, Yang Fang, Ping Zhang, Qiang Sun, Jiandong Li, Shiwen Wang

**Affiliations:** 1 National Key Laboratory of Intelligent Tracking and Forecasting for Infectious Diseases, NHC Key Laboratory of Medical Virology and Viral Diseases, National Institute for Viral Disease Control and Prevention, China CDC, Beijing 102206, China; liutiezhu1988@163.com (T.L.); yang_fang0326@163.com (Y.F.); sunqiang8611@126.com (Q.S.); 2 Department of General Surgery, Capital Center for Children’s Health, Capital Medical University, Beijing 100020, China; iwangxueqi@gmail.com; 3 Department of Pathology, Dezhou Second People’s Hospital, Dezhou 253000, China; zpdezhouey@163.com

**Keywords:** Dabie bandavirus, SNX11, V-ATPase, antiviral target, endosome

## Abstract

SNX11, a sorting nexin protein localized on the endosomal membrane, is an important protein closely related to protein sorting and endosomal trafficking. Previously, through a genome-wide CRISPR screening, we identified SNX11 as a critical protein for the entry of Dabie bandavirus. SNX11 deletion significantly inhibits the replication of Dabie bandavirus. We further discovered that the loss of SNX11 alters endosomal pH, potentially affecting the release process of Dabie bandavirus from endosomes to the cytoplasm. However, the mechanism by which SNX11 modulates endosomal pH and whether SNX11 deletion similarly inhibits other viruses remain to be elucidated. This study reveals that SNX11 can interact with the V1 subunit of the endosomal proton pump V-ATPase, affecting the expression level of this subunit on the endosomal membrane and thereby disrupting the assembly of V-ATPase. Additionally, we found that SNX11 deletion significantly inhibits the replication of dengue virus, hantavirus, and influenza virus. These findings suggest that SNX11 may be a key protein in the process of viral infection and could serve as a broad-spectrum antiviral target.

## 1. Introduction

Dabie bandavirus, belonging to the order *Bunyavirales*, family *Phenuiviridae*, and genus *Bandavirus* [1], was first discovered in 2010 in regions such as Henan and Hubei provinces, China [2]. It is transmitted through tick bites and can cause a newly emerging infectious disease known as Severe Fever with Thrombocytopenia Syndrome (SFTS) [3]. Since its discovery, cases have been reported in at least 28 provinces in China, including Hubei, Anhui, Jiangsu, and Liaoning provinces, with multiple localized outbreaks occurring [4,5]. In high-incidence provinces, the infection rate has shown a gradual upward trend in recent years [6]. Beyond mainland China, Dabie bandavirus infections have also been reported in several other countries and regions, such as Taiwan (China), South Korea, Japan, and Vietnam [4,7,8]. As a result, Dabie bandavirus has become a significant challenge to global public health [9,10].

To date, due to the fact that Dabie bandavirus does not induce significant cytopathic effects [11,12], there has been limited research on the screening of key factors involved in its infection [13]. In our previous study, we conducted an array-based CRISPR screening to identify host factors associated with the Dabie bandavirus infection. Ultimately, we identified eight host proteins, whose absence could inhibit the Dabie bandavirus infection to varying degrees, and notably the inhibitory effect was most pronounced in the absence of SNX11. At 96 h post-infection with Dabie bandavirus, the positive rate of viral nucleoprotein NP in SNX11-deficient cells decreased by 95% compared to the normal group [14].

Furthermore, we found that SNX11 deletion blocks the release of the virus from late endosomes into the cytoplasm, a process that hinges on the fusion of the viral envelope with the endosomal membrane. Subsequently, we discovered that the endosomal pH in SNX11-deficient HeLa cells (5.56) was 0.23 units higher than that in normal cells (5.33), suggesting that this pH alteration affects Dabie bandavirus membrane fusion. Given the previously mentioned role of SNX11 in the formation of acidic endosomal vesicles, we preliminarily hypothesize that SNX11 deficiency may block Dabie bandavirus release by altering endosomal pH [14]. However, the specific mechanism by which SNX11 regulates endosomal pH remains unclear, and to identify the proteins and mechanisms through which SNX11 modulates endosomal pH will provide important insights for discovering key targets of Dabie bandavirus and developing new antiviral drugs.

## 2. Material and Methods

### 2.1. Cell Culture and Virus Strains

HEK293T, A549, VERO, and MDCK cells, initially acquired from the American Type Culture Collection (ATCC; Manassas, VA, USA), were cultured in DMEM (Gibco, Grand Island, NY, USA) supplemented with 10% FBS (Gibco, Grand Island, NY, USA) and 1% penicillin/streptomycin (Gibco, Grand Island, NY, USA) at 37 °C/5% CO_2_. Cells were subcultured at 80–90% confluence using 0.25% trypsin-EDTA (Gibco, Grand Island, NY, USA) and authenticated via STR profiling. Dabie bandavirus (Strain HB29), Dengue virus 2 (DENV-2), Hantaan virus (Strain 76-118), and influenza A virus (H1N1; PR8) were used in the virus infection experiments. Viruses were propagated in Vero (DENV-2, DBV, HTNV) or MDCK (PR8) cells. Supernatants were clarified, aliquoted, and stored at −80 °C.

### 2.2. Generation of SNX11-Knockout 293T Cell Line Using CRISPR/Cas9

SNX11 knockout in human embryonic kidney 293T cells was achieved via CRISPR/Cas9 genome editing. Single-guide RNAs (sgRNAs), designed using the CHOPCHOP online tool to target distinct exons within the SNX11 gene (sgRNA sequence 5′-CACCGCCGCTACCGTGAGTTCGTG-3′ was cloned into the pSpCas9(BB)-2A-Puro (PX459) V2.0 vector (Addgene, Watertown, MA, USA). Cells were cultured in DMEM supplemented with 10% FBS and 1% penicillin/streptomycin at 37 °C with 5% CO_2_. Approximately 5 × 10^5^ cells were transfected with 2 µg of sgRNA plasmid or a control empty vector using Lipofectamine 3000, according to the manufacturer’s protocol. Transfected cells underwent puromycin selection (2 µg/mL) for 48 h post-transfection. Surviving cells were diluted and seeded for single-cell cloning. Genomic DNA from expanded monoclonal populations was extracted and the targeted regions amplified by PCR. Successful knockout was confirmed by Sanger sequencing analysis. Absence of SNX11 protein expression was further validated by Western blotting.

### 2.3. Quantification of Dabie bandavirus Viral RNA by RT-qPCR

To assess viral replication kinetics, wild-type (WT) and SNX11-KO 293T cells were infected with Dabie bandavirus with an original titer of 10^7^ TCID_50_/mL at a multiplicity of infection (MOI) of 0.1. Following a 1 h adsorption period at 37 °C, the inoculum was removed, cells were washed twice with PBS, and fresh complete medium was added. Cell culture supernatants were harvested at defined post-infection time points. Supernatants were clarified by centrifugation (500× *g*, 5 min) to remove cellular debris. Viral RNA was extracted from 140 µL of clarified supernatant using the QIAamp Viral RNA Mini Kit (Qiagen, Hilden, Germany), according to the manufacturer’s instructions. Reverse transcription-quantitative PCR (RT-qPCR) targeting a conserved region of the Dabie bandavirus L segment was performed.

### 2.4. RNA Sequencing and Bioinformatic Analysis

Total RNA from SNX11-KO and wild-type 293T cells (three biological replicates) was extracted. Libraries were prepared with Illumina TruSeq Stranded mRNA Kit and sequenced (150 bp paired-end) on NovaSeq 6000 (20 M reads/sample). Differentially expressed genes (DEGs) were identified with DESeq2 (v1.38.3; |log_2_FC| > 1, adj. *p* < 0.05, Benjamini–Hochberg). Pathway enrichment (KEGG 2023) and protein–protein interaction networks (STRING v12.0; confidence score > 0.7) were analyzed.

### 2.5. Co-Immunoprecipitation (Co-IP) Analysis of SNX11 and V-ATPase V1 Subunit Interaction

To investigate the interaction between SNX11 and the V-ATPase V1 subunit, HEK293T cells in 10 cm dishes were co-transfected with plasmids encoding HA-tagged SNX11 and FLAG-tagged V1 using Lipofectamine 3000 (Invitrogen, Carlsbad, CA, USA). Then, 48 h post-transfection, cells were washed with cold PBS and lysed on ice for 30 min in IP lysis buffer (50 mM Tris-HCl pH 7.4, 150 mM NaCl, 1% Triton X-100, 1 mM EDTA, protease inhibitors). Clarified lysates (14,000× *g*, 15 min, 4 °C) were incubated overnight at 4 °C with rotation with an anti-HA rabbit polyclonal antibody. Protein A/G agarose beads were added for 2 h. After five washes with lysis buffer, bound proteins were eluted by boiling in SDS-PAGE buffer. Eluates were subjected to SDS-PAGE, transferred to PVDF membranes, and probed with anti-FLAG (for V1) and anti-HA (for SNX11) mouse monoclonal antibodies, followed by HRP-conjugated secondary antibodies and ECL detection.

### 2.6. Pharmacological Inhibition of V-ATPase and Viral Replication Analysis

Wild-type (WT) and SNX11-knockout (SNX11-KO) HEK293T cells were pretreated with 100 nM Bafilomycin A1 (BafA1; V-ATPase inhibitor, Abcam, Cambridge, UK) or vehicle control (DMSO) for 1 h at 37 °C prior to infection with Dabie Bandavirus (MOI = 0.5). Cells were then infected for 1 h in the continuous presence of BafA1/DMSO, followed by removal of inoculum and maintenance in fresh medium containing corresponding treatments. At designated post-infection time points (0, 24, 48, 72 hpi), total RNA was extracted. RNA in supernatants of Dabie bandavirus was quantified using the AgPath ID one-step RT-PCR kit (ABI, Norwalk, CT, USA).

### 2.7. Subcellular Distribution Analysis of V-ATPase V1 Subunit

Plasma membrane and cytosolic fractions from SNX11-knockout (SNX11-KO) and wild-type (WT) HEK293T cells were isolated using the Minute™ Plasma Membrane Protein Isolation Kit (Invent Biotechnologies, Eden Prairie, MN, USA). Cells were lysed in hypotonic buffer, followed by differential centrifugation: 700× *g* for 10 min (nuclear removal) and 100,000× *g* for 1 h at 4 °C to separate cytosolic (supernatant) and total membrane (pellet) fractions. Membrane pellets were solubilized in RIPA buffer with protease inhibitors. Equal protein amounts (20 μg) from each fraction were resolved by SDS-PAGE and transferred to PVDF membranes. Blots were probed with Mouse anti-V-ATPase V1 subunit antibody (1:1000; Proteintech, Rosemont, IL, USA) and Rabbit β-actin (1:2000; cell signaling technology, Danvers, MA, USA). HRP-conjugated secondary antibodies and ECL were used for detection.

### 2.8. Confocal Microscopy

Cells in 8-chamber slides were fixed with 4% PFA (15 min, RT), permeabilized with 0.1% Triton X-100 (10 min), and blocked with 5% BSA (1 h). Primary antibodies were incubated overnight at 4 °C: rabbit anti-SNX11 (1:200) and mouse anti-V-ATPase V1 subunit (1:500). After PBS washes, samples were treated with secondary antibodies for 1 h (RT): goat anti-rabbit IgG-Alexa Fluor 488 (1:500) and goat anti-mouse IgG-Alexa Fluor 594 (1:500). Nuclei were stained with DAPI (0.1 µg/mL, 5 min). Slides were imaged using a laser-scanning confocal microscope (Leica TCS SP8, Wetzlar, Germany) with a 63× oil objective. Image processing used the manufacturer’s software.

### 2.9. Validation of SNX11 Deficiency on Infection by Diverse Viruses

To determine whether the inhibitory effect of SNX11 deficiency extends beyond Dabie bandavirus, SNX11-knockout (SNX11-KO) and wild-type (WT) 293T cells were infected with dengue virus serotype 2 (DENV-2). Viral infection was assessed at 48 h post-infection (hpi) by measuring the levels of DENV-2 NS1 antigen released into the culture supernatant using a commercial ELISA kit (MEIMIAN, Taizhou, China). Additionally, infection with the Hantaan virus strain 76-118 (HTNV-76-118) and influenza A/Puerto Rico/8/1934 (H1N1; PR8) was assessed using the newly constructed SNX11-KO A549 cell line alongside their corresponding WT A549 controls. Similarly, at 48 hpi, viral infection for both HTNV-76-118 and PR8 was quantified by ELISA, targeting the viral nucleoprotein (N protein) present in the infected cell supernatants.

### 2.10. Statistical Analysis

Statistical analyses were performed using GraphPad Prism 9.0. Data are presented as mean ± standard deviation (SD). For comparisons between two groups, unpaired two-tailed Student’s *t*-tests were applied. RNA-seq differential expression was analyzed with DESeq2 (adj. *p* < 0.05, |log_2_FC| > 1). ELISA/qPCR data derived from ≥3 biological replicates (each with triplicate technical repeats). Power analysis confirmed ≥80% power for key experiments at α = 0.05.

## 3. Results

**Figure 1:**
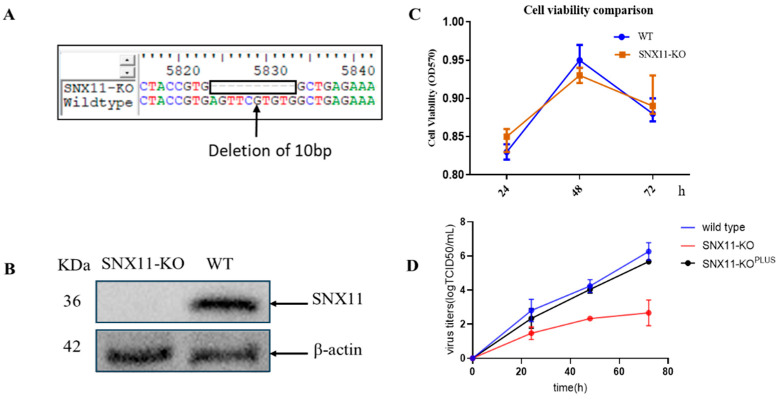
Generation and validation of SNX11-knockout (SNX11-KO) 293T cells and their resistance to the Dabie bandavirus infection. (**A**) Sanger sequencing of the SNX11 locus in wild-type (WT) and SNX11-KO 293T cells. Arrows indicate deletion (indel) mutations causing frameshifts. (**B**) Western blot analysis of SNX11 protein expression in wild-type (WT) and SNX11-KO 293T cells. β-actin served as a loading control. (**C**) Cell proliferation assay (CCK-8) showing no significant difference in viability between WT and SNX11-KO cells over 72 h. (**D**) Dabie bandavirus infection efficiency in WT vs. SNX11-KO cells. Viral RNA in supernatants was quantified by RT-qPCR.

In our preliminary investigations, HeLa cells were initially employed for CRISPR screening and validation procedures owing to their experimental robustness and operational convenience [15]. Although 293T cells demonstrated reduced compatibility with CRISPR screening platforms compared to HeLa cells, their superior susceptibility to Dabie bandavirus infection coupled with enhanced transfection efficiency made them more appropriate for subsequent functional characterization. Based on these considerations, we generated SNX11-knockout 293T cell lines using the CRISPR/Cas9 system in the current study. The efficiency of protein depletion was confirmed by Western blotting (Figure 1A,B).

Quantitative assessment of cellular proliferation kinetics through CCK-8 assays revealed comparable growth profiles between wild-type and SNX11-depleted cells (*p* > 0.05), confirming that SNX11 ablation does not compromise 293T cell proliferative capacity (Figure 1C). To delineate the antiviral mechanism, isogenic cell models including SNX11 knockout cells and their SNX11-reconstituted counterparts were challenged with Dabie bandavirus. Longitudinal monitoring of viral RNA dynamics in culture supernatants via RT-PCR demonstrated a reduction in viral RNA load in knockout cells relative to controls. This phenotype was effectively rescued upon SNX11 re-expression, providing orthogonal validation that SNX11 depletion exerts significant inhibitory effects on Dabie bandavirus replication (Figure 1D).

**Figure 2:**
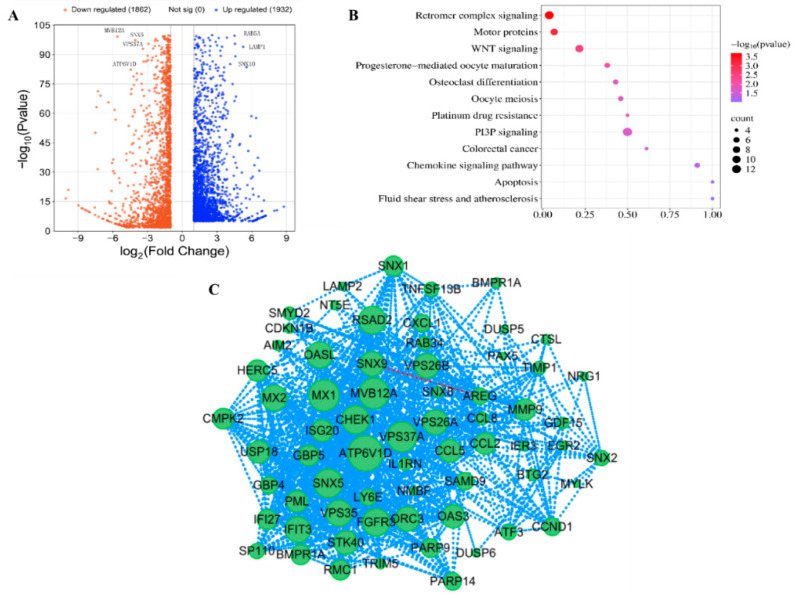
Transcriptomic profiling reveals SNX11 knockout-induced signaling rewiring in 293T cells. (**A**) Volcano plot of differentially expressed genes (DEGs) in SNX11-knockout (SNX11-KO) vs. wild-type (WT) 293T cells. (**B**) KEGG pathway enrichment of DEGs. Top 12 significantly altered pathways (ranked by enrichment *p* value < 0.05) are shown. Dot size reflects gene count; color scale indicates −log10(*p* value). (**C**) Protein–protein interaction (PPI) network of high-confidence DEG-encoded protein.

To investigate the intracellular function of SNX11, we performed transcriptome sequencing to analyze transcriptomic alterations in cells before and after SNX11 knockout. The results revealed that SNX11 deficiency resulted in the downregulation of 1862 proteins, including key regulators of intraluminal vesicle formation and endosomal pH (ATP6V1D, MVB12A, VPS37A). Conversely, it induced the upregulation of 1932 proteins, such as essential endosomal trafficking factors RAB5A and LAMP1 (Figure 2A). SNX11 depletion predominantly perturbed signaling pathways associated with retromer complex signaling and PI3P signaling (Figure 2B).

PPI network analysis revealed that among the differentially expressed proteins resulting from SNX11 deficiency, vesicular trafficking proteins such as ATP6V1D, VPS16, VPS26A, VPS26B, and VPS35 were positioned within the core module of the interaction network. This finding further underscores the functional association of SNX11 with vesicular acidification regulation (exemplified by ATP6V1D) and intracellular trafficking (Figure 2C). Notably, the close functional interplay between SNX11 and V-ATPase was further corroborated by this network.

**Figure 3:**
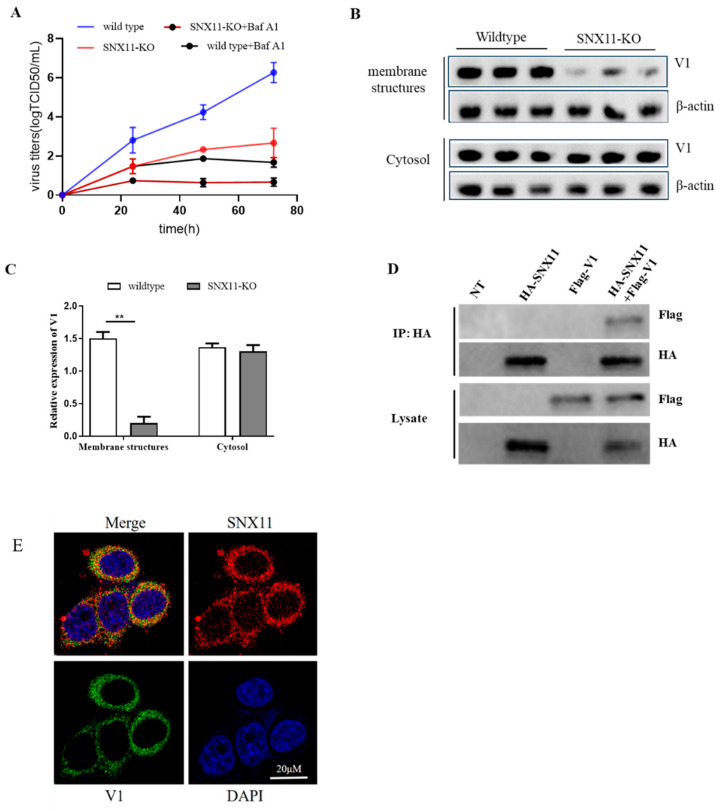
SNX11 regulates V-ATPase assembly and promotes Dabie bandavirus entry via V1 subunit interaction. (**A**) Bafilomycin A1 (Baf A1) inhibition assay: viral RNA in supernatants after Dabie bandavirus infection. (**B**) Plasma membrane fractionation: immunoblot of V1 subunits in membrane fractions and cytosol of WT vs. SNX11-KO cells. (**C**) Quantification of V1 subunit expression: reduced V1A levels in SNX11-KO membrane fractions (n = 3; ** *p* < 0.01, unpaired *t*-test). (**D**) Co-immunoprecipitation (Co-IP): SNX11-HA immunoprecipitates with V1A in 293T cells. Lanes: input (10%). (**E**) Confocal microscopy: colocalization of SNX11 (red) and V1 (green) in perinuclear regions, scale bars: 20 μm.

To determine whether Dabie bandavirus infection relies on V-ATPase activity, we treated both SNX11-knockout (SNX11-KO) and wild-type 293T cells with Bafilomycin A1, a V-ATPase inhibitor, prior to Dabie bandavirus infection. Viral supernatants were collected at various post-infection time points and subjected to quantitative RT-PCR (qRT-PCR) analysis. Notably, viral RNA levels exhibited a significant reduction in both cell types treated with the inhibitor compared to untreated controls, and the viral RNA level were lower in SNX11-KO cells, demonstrating a critical role of V-ATPase in the Dabie bandavirus infection (Figure 3A).

To investigate whether SNX11 depletion alters V-ATPase localization on endosomal membranes, thereby impairing endosomal pH regulation, we performed plasma membrane isolation and Western blotting to detect changes in V-ATPase subunit expression before and after SNX11 knockout. Our results revealed that SNX11 deficiency significantly reduced the expression of the V1 subunit on endosomal membranes (Figure 3B,C). To explore potential interactions between SNX11 and V-ATPase, we conducted co-immunoprecipitation (co-IP) experiments, which demonstrated that SNX11 specifically interacts with the V1 subunit of V-ATPase (Figure 3D). Supporting these findings, laser confocal microscopy analysis revealed substantial spatial colocalization between the two molecules (Figure 3E). These findings provide compelling evidence that SNX11 regulates endosomal pH through its interaction with V-ATPase.

**Figure 4:**
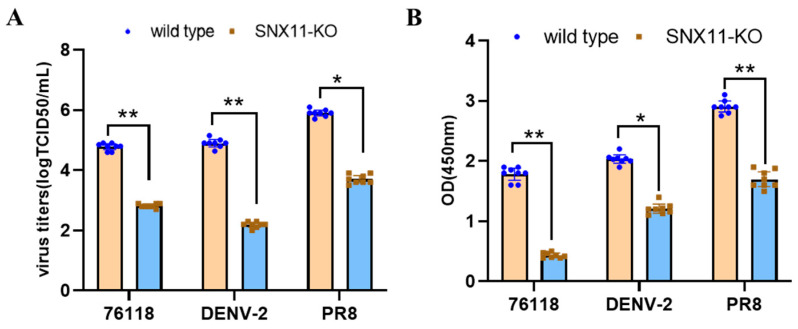
SNX11 deficiency broadly inhibits viral infection. (**A**) Viral RNA levels in supernatants of SNX11-knockout (SNX11-KO) and control cells infected with Dengue virus (DENV; 293T cells), Hantaan virus (HTNV 76-118; A549 cells), or influenza virus (PR8; A549 cells), quantified by qRT-PCR at 48 h post-infection (hpi). (**B**) Viral antigen load in supernatants of infected SNX11-KO and control cells, measured by ELISA at 48 hpi. * Data represent mean ± SD (n = 8 independent experiments). ** *p* < 0.01, SNX11-KO: SNX11-knockout.

**Figure 5:**
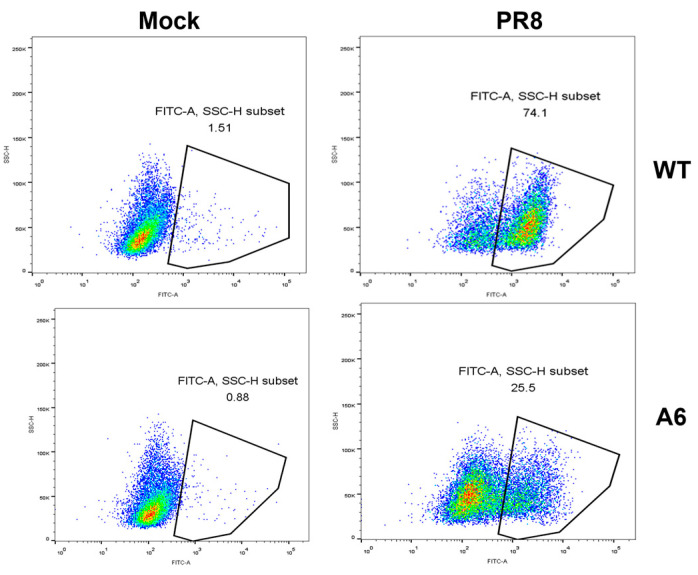
SNX11 deficiency broadly inhibits the influenza virus infection. A6 represents SNX11-KO A549 cell line.

To validate the broad-spectrum nature of SNX11 deficiency in suppressing viral replication, we infected the aforementioned SNX11-deficient 293T cells and wild-type cells with dengue virus and utilized previously generated SNX11-deficient A549 cells [15] and wild-type cells to infect the Hantaan virus strain 76-118 (HTNV 76-118) and the influenza virus PR8 strain. At 48 h post-infection, quantification of viral RNA levels and viral antigen load in cell supernatants, including the detection of Dengue virus NS1 antigen, PR8 strain NP antigen, and HTNV 76-118 NP antigen, revealed that SNX11 deficiency significantly suppressed the replication of Dengue virus, hantavirus, and influenza virus, as shown in Figure 4A,B. Furthermore, flow cytometric analysis of influenza PR8-infected SNX11-KO and wild-type A549 cells demonstrated that SNX11 deficiency markedly reduced the viral antigen-positive cell population from 74.1% to 25.5% (Figure 5). The quantification of these specific viral antigens further supports the conclusion that SNX11 depletion exerts a potent inhibitory effect against diverse viral families.

## 4. Discussion

Structurally, V-ATPase consists of two functional domains: the membrane-embedded V0 complex, which mediates proton transport, and the cytoplasmic V1 complex, which hydrolyzes ATP to energize this process [16,17]. This enzyme is indispensable for maintaining the acidic luminal pH of lysosomes and endosomes as well as modulating the pericellular microenvironment in specialized cells [18,19]. V-ATPase mechanistically regulates endosomal pH through two interconnected pathways, primarily by modulating the dynamic equilibrium between V0 and V1 subunit dissociation and assembly, which directly governs its proton-pumping activity [20,21]. Additionally, through controlling the membrane abundance of V0 and V1 subunits, it determines the density of functional proton-transporting complexes on endosomal membranes [22]. These coordinated processes collectively establish the acidic endosomal environment essential for intracellular trafficking [23].

SNX11, a 264-amino acid member of the SNX family, features an N-terminal lipid-binding motif and two C-terminal α-helical domains [24]. We previously identified SNX11 as a critical host factor required for the cellular entry of Dabie bandavirus, with initial evidence suggesting its involvement in the viral endocytic pathway. However, the precise mechanism by which SNX11 facilitates viral entry remained unclear. In our previous attempts to investigate potential interactions between SNX11 and Dabie bandavirus proteins using co-immunoprecipitation (co-IP) assays, we did not detect any interaction between SNX11 and viral proteins. This suggests that SNX11 likely facilitates infection indirectly via host pathway modulation rather than through direct virion binding. This study demonstrates for the first time that SNX11 interacts with the V1 subunit of V-ATPase, regulating its trafficking to endosomal membranes. This interaction modulates endosomal pH, ultimately affecting the endocytic entry of Dabie bandavirus. This conclusion provides a new perspective for investigating viral endocytosis and expands the functional repertoire of the SNX protein family.

While our study establishes SNX11’s role in v-ATPase assembly and endosomal acidification, broader functions of SNXs in viral infection warrant consideration. SNXs typically regulate viral entry through three primary mechanisms: SNXs can bind viral envelope proteins, mediating viral trafficking from early to late endosomes [25,26]; SNXs interact with viral nucleoproteins to facilitate membrane fusion and uncoating processes [27]; SNXs regulate actin cytoskeleton dynamics by binding microfilament-associated proteins, thereby modulating intracellular viral transport through actin depolymerization/polymerization [28]. Notably, prior characterization of SNX11 has been limited to TRPV3 degradation [29] and functional interplay with SNX10 in endosomal acidification. Our findings now position SNX11 as a central coordinator of endosomal maturation required for diverse viral life cycles.

To validate the broad impact of SNX11 deficiency on viral entry, we selected phylogenetically diverse viruses: Hantaan virus (segmented negative-sense RNA virus like Dabie bandavirus [30,31]), influenza virus (segmented negative-sense RNA virus [32]), and Dengue virus (positive-sense single-stranded RNA virus [33,34]). SNX11 knockout consistently inhibited infection by all tested viruses. This pan-viral suppression can be interpreted through dual mechanisms. Primarily, as a sorting nexin (SNX) family member that intrinsically regulates endosomal trafficking of cellular cargoes [35,36,37], SNX11 ablation would be expected to disrupt endosomal transport of viruses. However, our earlier genome-wide CRISPR screen uniquely identified SNX11 among all SNX family members as an essential host factor for viral entry, suggesting its distinctive role as a specific regulator of viral endocytosis. Future investigations will focus on delineating the precise mechanisms underlying SNX11-virus interactions.

## Data Availability

The original contributions presented in the study are included in the article, further inquiries can be directed to the corresponding author.
